# Valuable Prescription for Neuralgia

**Published:** 1902-09

**Authors:** N. F. Howard

**Affiliations:** Dahlonega, Ga.


### CORRESPONDENCE.

### VALUABLE PRESCRIPTION FOR NEURALGIA.

DAHLONEGA, GA., August 22, 1902.

*Editor Atlanta Journal-Record of Medicine:*

| | |
|---|---|
| Muriate of ammonia | ℥iii. |
| Tincture of belladonna | ʒi. |
| Tincture of hyoscyamus | ʒii. |
| Aqua menthæ piperitæ | ℥iv. |
| Aqua camphoræ | ℥x |

or a sufficient quantity to make 16 ounces of the mixture.

Mix.

The dose of this medicine is from one half to one teaspoonful, given in a little sweetened water, every two or three hours.

Every drachm of this preparation contains eleven grains of muriate of ammonia.

This medicine was a great favorite of Dr. Watson for hemicrania, for which he gave five, ten, to thirty grains every three hours, as stated in his lectures. He does not, however, mention the efficacy of the medicine in any other form of neuralgia.

We have found by the use of this medicine since we began the practice of medecine, that it relieves pain in other forms of neuralgia as well as in hemicrania.

We desire to say, that for more than twenty years, we have used, with the most gratifying results, in all phases of neuralgia, the above formula, which we here send you for publication.

If muriate of ammonia will relieve hemicrania, why then will it not relieve all forms of neuraliga? In my opinion, it will. Being of this opinion, I have given it in many forms of neuralgia with entire success, as hemicrania, gastralgia, pleurodynia, sciatica, and other pains that simulate neuralgia, especially in the joints.

I had some doubt about sciatica that occurred in my own person, some months ago, when I used my formula, and received great relief from the same. I give another case that occurred in my own person, some two weeks ago, from cold and exposure; I had pain in my right eye, my right brow, my eye-tooth, and my ear, but suffered in no other part of my head. I was led to make this inquiry, Is this hemicrania or is it sun-pain? After suffering from the pain for some time I began the use of the neuralgic preparation, of which I took a half teaspoonful every two or three hours, and in twenty-four hours the pain was almost entirely relieved, and fully well in another twenty-four hours.

I trust your valuable journal will give me a place for this communication.

N. F. HOWARD, M.D.

---

THE following are extracts from a bulletin just given out by the State Agricultural Department of Minnesota: The opinion entertained by a great many people that whole wheat and Graham bread is more nutritious than that made from standard patent flour, the flour used in every-day baking, is erroneous. When milk was used as a ration with bread, butter, beans, eggs, and potatoes, all the protein of the milk was digested, and in addition 4.91 per cent. more of the protein of the other foods with which it was combined was digested than when the milk was omitted. The highest degree of digestibility was secured in a mixed ration. Experiments made with butter showed that it has a high degree of digestibility, 98 per cent. of it being available to the body. Cheese should be used in the diet regularly and in small quantities, rather than at irregular intervals and in large quantities, as is frequently the case. Cheese ordinarily is one of the cheapest and most nutritious foods that can be procured. It is possible to secure a larger amount of digestible nutrients and available energy from cheese costing 15 cents a pound than from meat costing 10 cents a pound. Oatmeal, like cheese and beans, is slow of digestion, requiring much intestinal work for the digestive process; but if well prepared and thoroughly cooked it is a suitable food for persons of all habits.

